# Unraveling the Cycle: A Scoping Review Exploring the Impact of Antidepressants on the Female Reproductive Cycle

**DOI:** 10.7759/cureus.97360

**Published:** 2025-11-20

**Authors:** Shannon Weatherly, Carly D Garazi, Emma Woldenberg, Trisha Ravigopal, Joshua Costin

**Affiliations:** 1 Osteopathic Medicine, Dr. Kiran C. Patel College of Osteopathic Medicine, Fort Lauderdale, USA; 2 Medical Education, Nova Southeastern University, Dr. Kiran C. Patel College of Allopathic Medicine, Fort Lauderdale, USA

**Keywords:** antidepressant drug, bupropion, female reproductive health, fluoxetine, hormonal regulation, menstrual cycle changes, sertraline, snri’s, ssri’s, venlafaxine

## Abstract

Antidepressants, particularly selective serotonin reuptake inhibitors (SSRIs) and serotonin-norepinephrine reuptake inhibitors (SNRIs), are widely prescribed for mood disorders, including in individuals of reproductive age. While their psychiatric effects are well-documented, emerging evidence suggests these medications may influence hormonal regulation, ovulation, and menstrual cycle patterns. Potential mechanisms include disruption of the hypothalamic-pituitary-ovarian (HPO) axis, alterations in serotonergic signaling, and medication-specific hormonal fluctuations. These interactions raise important questions about the understudied reproductive impact of commonly prescribed antidepressants.

In accordance with Preferred Reporting Items for Systematic Reviews and Meta-Analyses Extension for Scoping Reviews (PRISMA-ScR) guidelines, a comprehensive search of Ovid MEDLINE, Excerpta Medica Database (EMBASE), and Cumulative Index to Nursing and Allied Health Literature (CINAHL) databases were performed to identify studies examining the effects of antidepressants on ovarian function, menstrual regularity, ovulation, or hormone levels. Eligible studies included those involving individuals assigned female at birth (AFAB) of reproductive age, as well as animal models with comparable reproductive physiology. Both human and preclinical studies were considered, covering a range of antidepressant classes: SSRIs, SNRIs, atypical antidepressants, tricyclic antidepressants (TCAs), monoamine oxidase inhibitors (MAOIs), and newer agents such as vortioxetine and vilazodone.

Out of 34 eligible studies, 16 were included in the final synthesis. SSRIs, especially fluoxetine, sertraline, paroxetine, escitalopram, and citalopram, were most frequently studied. Observational studies reported increased rates of menstrual irregularity and sexual dysfunction, particularly with chronic SSRI use. Antidepressant use was associated with reduced fecundability, even after adjusting for depression severity. Bupropion, venlafaxine, and other SNRIs showed cycle-phase-dependent pharmacokinetics and variable hormonal interactions. Antidepressant use was associated with changes in menstrual cycle length and increased cardiometabolic risk; however, they may normalize low testosterone levels in depressed women, with improvements in sexual function post-treatment. Clinical and preclinical findings indicate that SSRIs may impair ovulation through serotonergic inhibition of gonadotropin-releasing hormone (GnRH) as well as downstream suppression of luteinizing hormone (LH) and follicle-stimulating hormone (FSH), with some studies also noting elevated prolactin and follicular atresia. Taken together, these findings suggest a biologic basis for the observed menstrual irregularities and reduced fecundability reported in observational and cohort studies.

Across studies, reproductive effects varied by antidepressant class, duration of use, and underlying mood pathology. Observed trends support a drug- and class-dependent impact on estrogen, progesterone, prolactin, and reproductive function, with additional implications for systemic health.

## Introduction and background

Antidepressants are now integral to the management of mood and anxiety disorders in the United States. Between 2015 and 2018, 13.2% of adults reported antidepressant use in the preceding 30 days, with prevalence nearly twice as high in women (17.7%) as in men (8.4%) [1]. Among reproductive-aged, nonpregnant women, roughly 5% meet criteria for major depressive disorder, and one-third of these patients take an antidepressant, with selective serotonin reuptake inhibitors (SSRIs) accounting for 21.3% of prescriptions [2]. SSRIs and serotonin-norepinephrine reuptake inhibitors (SNRIs) are first-line pharmacotherapy for major depressive and generalized anxiety disorders and rank among the most frequently dispensed drug classes nationwide [3].

Women's greater lifetime risk of depression is thought to reflect interactions between mood regulation and the pronounced hormonal fluctuations of the reproductive cycle [4]. Neuroendocrine pathways controlling mood and reproduction converge within the hypothalamic pituitary gonadal axis, and rapid shifts rather than absolute levels of estrogen and progesterone appear most closely linked to mood vulnerability during the luteal phase, postpartum period, and menopausal transition [4]. These same fluctuations may interact bidirectionally with antidepressant therapy, yet the influence of these drugs on menstrual physiology and reproductive hormone dynamics remains poorly defined.

This susceptibility appears to be more than psychosocial in origin and may reflect a biological interplay between antidepressant mechanisms and reproductive endocrinology. SSRIs and SNRIs exert therapeutic effects through serotonergic and noradrenergic modulation, pathways that overlap with hypothalamic regulators of gonadotropin-releasing hormone pulsatility and downstream ovarian function. Preclinical studies demonstrate that serotonergic perturbations can disrupt luteinizing hormone surges and interfere with ovulatory timing [5, 6]. Clinically, menstrual irregularities and hormonal disturbances have been reported during SSRI and SNRI therapy, but systematic investigation of these effects remains limited.

SSRIs and SNRIs represent the cornerstone of contemporary pharmacotherapy, while other classes, including tricyclic antidepressants (TCAs) and newer atypical agents such as bupropion, mirtazapine, and vortioxetine, offer important alternatives. These medications broadly enhance monoaminergic signaling, though they differ in their molecular targets and downstream effects. SSRIs selectively inhibit the serotonin transporter (SERT), preventing reuptake of 5-hydroxytryptamine (5-HT) into presynaptic neurons, thereby enhancing serotonergic neurotransmission [7]. SNRIs inhibit the reuptake of both serotonin and norepinephrine by blocking their respective transporters, increasing synaptic concentrations of these monoamines [8]. Atypical antidepressant agents such as vortioxetine exert mixed mechanisms, including norepinephrine-dopamine reuptake blockade (bupropion), α2-adrenergic antagonism with 5-HT2/5-HT3 blockade (mirtazapine), and multimodal serotonergic activity [8]. Monoamine oxidase inhibitors (MAOIs) irreversibly inhibit monoamine oxidase enzymes, MAO-A and MAO-B, which degrade serotonin, norepinephrine, and dopamine, thereby increasing synaptic levels of all three [8].

Although the psychiatric efficacy of antidepressants is well established, their endocrine sequelae are not well defined. Sexual dysfunction remains the most consistently reported adverse effect, with diminished libido, delayed or absent orgasm, and reduced sexual satisfaction affecting up to 70% of serotonergic antidepressant users [9]. Risk factors for these side effects are both dose-dependent and molecule-specific, with paroxetine carrying the greatest burden [9]. They are proposed to act through serotonin-mediated suppression of dopaminergic tone, prolactin release, and inhibition of nitric oxide synthase; however, definitive pathways have yet to be confirmed [10].

Despite their clinical utility, antidepressants engage neural and endocrine networks that remain only partially mapped, underscoring the need to clarify how these drugs influence broader physiologic systems, particularly those involved in reproductive regulation. Normal menstrual cyclicity depends on the integrity of the hypothalamic-pituitary-ovarian axis. Pulsatile gonadotropin-releasing hormone from the hypothalamus drives luteinizing hormone and follicle-stimulating hormone release, coordinating follicular development, ovulation, and steroidogenesis [11]. Estradiol and progesterone, in turn, modulate serotonergic and noradrenergic activity within corticolimbic regions implicated in mood regulation [12, 13]. Serotonin can alter gonadotropin-releasing hormone pulsatility [9], and experimental manipulation of serotonergic or noradrenergic tone disrupts luteinizing hormone surges and ovulatory timing [6, 14]. These observations provide a biologically plausible link between antidepressant pharmacology and menstrual disruption.

Clinical data lend further support: a population-based study found menstrual disorders in 24.6% of antidepressant users versus 12.2% of matched controls, with an incidence of 14.5% after drug initiation; paroxetine, sertraline, venlafaxine, and regimens that included mirtazapine were most frequently implicated [15]. Nevertheless, evidence remains limited. Randomized trials rarely include endocrine or menstrual endpoints, many observational studies lack hormonal assays, and concurrent oral-contraceptive use, which can confound or mask medication effects, is inconsistently recorded. Depressive illness itself can suppress the hypothalamic-pituitary-ovarian axis, further complicating causal interpretation [16, 17].

Given these biologically plausible yet insufficiently characterized intersections, this scoping review synthesizes current evidence on the impact of antidepressants on cycle length, ovulatory function, and reproductive hormone profiles. Mapping clinical, mechanistic, and pharmacokinetic findings will clarify existing knowledge, highlight research gaps, and inform future investigations focused on psychopharmacology and female reproductive health. As use of antidepressants expands in this demographic, elucidating their effects on reproductive physiology remains a critical step toward optimizing both psychiatric and gynecologic outcomes.

## Review

Methods

Objective

This scoping review methodology was selected to support a comprehensive synthesis and mapping of current evidence regarding the reproductive effects of antidepressant use, with particular focus on menstrual cycle regulation, ovarian function, and hormonal dynamics in both human and animal models. By identifying key findings, knowledge gaps, and emerging mechanistic pathways, such as serotonergic signaling and hypothalamic-pituitary-ovarian (HPO) axis disruption, this review aims to inform future research directions and clinical understanding of how commonly prescribed antidepressants may impact reproductive health. The review was conducted in accordance with the Joanna Briggs Institute (JBI) Review Manual [18] and followed the Preferred Reporting Items for Systematic Reviews and Meta-Analyses Extension for Scoping Reviews (PRISMA-ScR) guidelines [19].

This review was carried out using the Population, Concept, Context (PCC) model. The population included humans assigned female at birth (AFAB) of reproductive age, as well as non-human mammals with comparable reproductive physiology. Studies examining individuals outside of the typical reproductive age range were also considered when they provided mechanistic insights or involved comparable hormonal profiles. To assess the concept within these populations, eligible studies assessed outcomes related to ovarian function, menstrual-cycle characteristics (e.g., cycle length, variability, and regularity), hormonal regulation (via follicle-stimulating hormone (FSH), luteinizing hormone (LH), estradiol, progesterone, prolactin), puberty onset, follicular development, or menstrual irregularities. Mechanistic studies targeting the HPO axis, serotonergic signaling, gonadotropin release, ovarian receptor expression, or steroidogenesis were also included. The context included both clinical and preclinical investigations. Clinical studies encompassed randomized and non-randomized trials, observational studies (cohort, cross-sectional, and case-control), case series, individual case reports, and secondary analyses of prospective fertility or reproductive health datasets. Preclinical studies included *in vivo* animal investigations with translational relevance. Review articles that synthesized primary data were also included. Only peer-reviewed publications in English (or translated into English) from 2010 to 2025 were considered.

Search Strategy Details

The literature search was conducted on February 2, 2025, with two rounds of independent, blinded review by study authors to ensure consistency in screening and selection. Eligible study designs included clinical trials, observational studies, case series, case reports, preclinical in vivo investigations, and review articles synthesizing primary data. This scoping review utilized the Excerpta Medica Database (EMBASE), Ovid MEDLINE, and the Cumulative Index to Nursing and Allied Health Literature (CINAHL) databases to identify relevant studies. The key search terms included: "antidepressants" OR "antidepressant agents" OR "psychotropic drugs" AND "reproductive cycle" OR "menstrual cycle" OR "hormonal fluctuations" OR "ovulation" OR "cycle regularity".

Studies were included if they were original, peer-reviewed publications examining the effects of antidepressant use on reproductive or hormonal outcomes in humans AFAB of reproductive age, or in non-human mammalian models with comparable physiology. Eligible works also encompassed studies that reported mechanistic insights into the HPO axis, serotonergic signaling, gonadotropin release, or ovarian physiology in relation to antidepressant exposure. Clinical trials, observational studies, case series, case reports, preclinical in vivo investigations, and review articles synthesizing primary data were all eligible for inclusion.

Studies were excluded if they focused exclusively on post-menopausal populations without relevant mechanistic data; on pregnant, postpartum, or breastfeeding individuals without broader implications for reproductive function; on non-mammalian species; or on in vitro models without in vivo applicability. Trials investigating non-antidepressant treatments were omitted unless they provided comparative reproductive data alongside antidepressant exposure. Papers limited to mental-health outcomes, quality of life, or neurotransmission without reproductive endpoints were excluded. Studies published in languages other than English, along with book chapters, editorials, non-peer-reviewed reports, and opinion pieces, were excluded. 

Quality Assessment

The articles selected after two independent rounds of blind screening were evaluated for quality using the JBI critical appraisal tools [18]. Each article was evaluated by each author, and discrepancies between the scoring of the evaluators were discussed and resolved, with three authors ultimately agreeing upon a checklist score. Studies were included if they had a low risk of bias or better, defined as a score of 70% or above following the JBI critical appraisal method.

Data Extraction, Management, and Analysis

For managing records and data throughout the review, Sumari (JBI, Adelaide, Australia) and Excel (Microsoft, Redmond, WA) software programs were utilized. Data was extracted from papers included in the scoping review by two or more independent reviewers into an Excel spreadsheet. Extracted data included participant information, the core concept, context, study methods, and any other pertinent key findings. After conducting the data analysis, a PRISMA flow chart was utilized to track the steps followed in the search strategy.

Results

**Figure 1:**
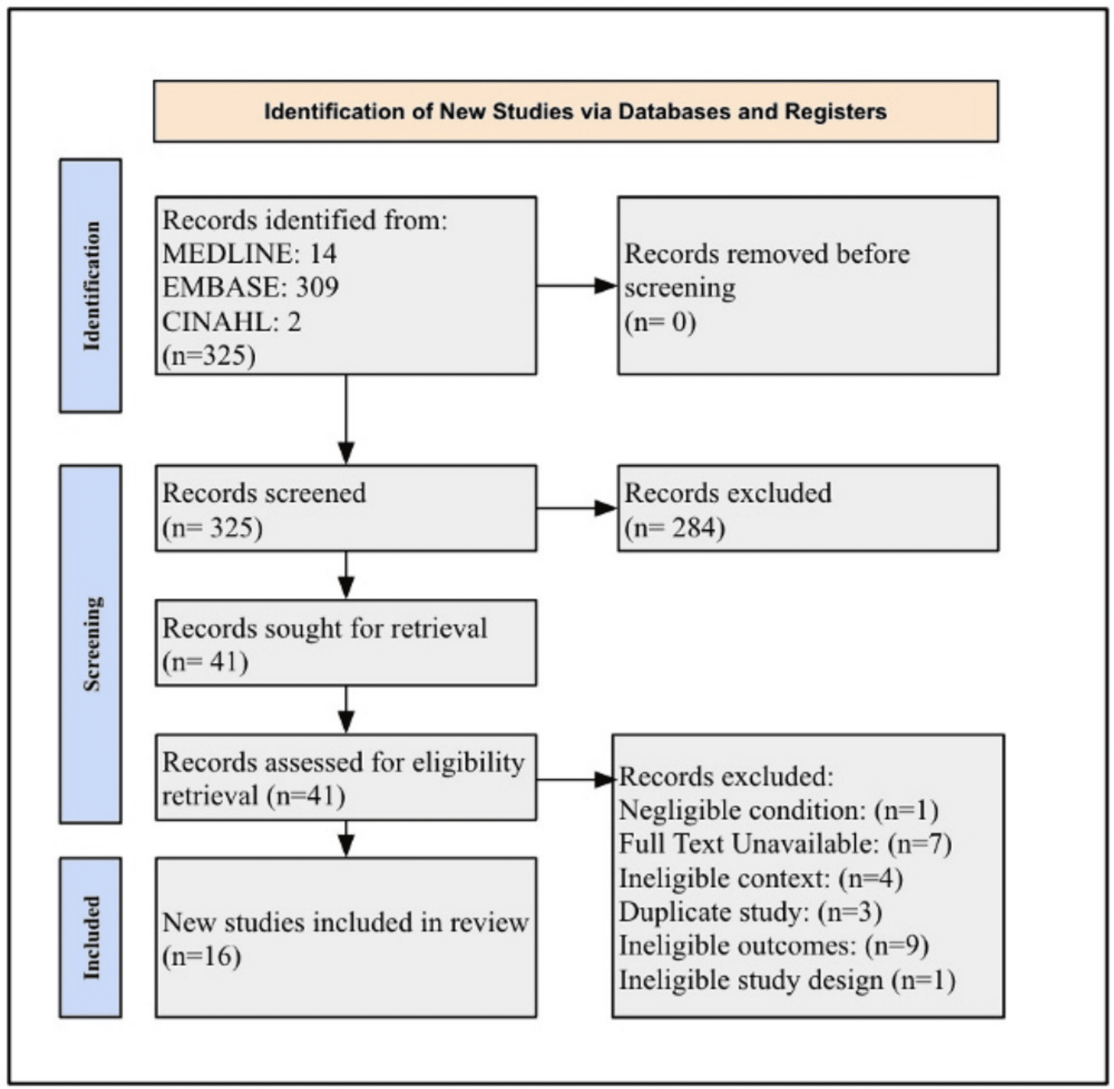
Preferred Reporting Items for Systematic Reviews and Meta-Analyses Extension for Scoping Reviews (PRISMA-ScR) flow diagram PRISMA-ScR flow diagram outlining the selection of articles for inclusion in the present review

After searching the databases using the pre-determined search terms and applying the inclusion and exclusion criteria, 16 articles were identified as being within the scope of this review (Figure 1). Data was extracted from all 16 included articles and can be referenced in Table 1. All included studies were assessed as having a low risk for bias according to the JBI critical appraisal tools [18]. 

**Table 1:** Data extraction chart. Data was extracted from each of the articles included in this review and listed in this table for direct comparison HDL - high-density lipoprotein; SSRI - selective serotonin reuptake inhibitors; GnRH - gonadotropin-releasing hormone; TCA - tricyclic antidepressants; NFLU - norfluoxetine; ACTH - adrenocorticotropic hormone; cAMP - cyclic adenosine monophosphate; ATP - adenosine triphosphate; FLX - fluoxetine; SNRI - serotonin-norepinephrine reuptake inhibitors

Reference	Study design	Study findings	Antidepressant class	Antidepressant type
Bleil et al. 2013 [20]	Observational study (cross-sectional)	The study analyzed 804 premenopausal women (aged 25-45) from a multi-ethnic cohort recruited through Kaiser Permanente Northern California. Participants had regular menstrual cycles, intact ovaries/uterus, and no major medical illnesses. This study found that changes in menstrual cycle length among healthy premenopausal women were associated with higher cardio-metabolic risk, lower HDL levels, and greater depression severity, diagnosis, and antidepressant use. Exploratory analyses suggested that menstrual disruptions may partially mediate the relationship between depression and cardio-metabolic risk, indicating a potential shared biological pathway. These findings support the idea that subtle ovarian changes may serve as early markers of both reproductive and cardiovascular health risks.	Unspecified	Unspecified
Çam and Bilgin 2015 [21]	Case report	This case of a 34-year-old female patient, highlights bupropion-associated galactorrhea and hyperprolactinemia, likely due to dopamine receptor downregulation rather than increased dopamine activity. Symptoms resolved rapidly within four days of discontinuing bupropion, with normalization of prolactin levels, supporting a strong causal link. The patient's concurrent sertraline use was deemed unlikely to be the cause, given the typical persistence of SSRI-induced hyperprolactinemia.	Atypical	Bupropion
Casilla-Lennon et al. 2016 [22]	Observational study (cohort study)	This study looked at 957 women aged 30-44 years with no history of infertility, trying to conceive and found that antidepressant use, particularly SSRIs, was associated with reduced fecundability in women trying to conceive, even after adjusting for a history of depression. The findings suggest the effect may stem from antidepressant-induced suppression of GnRH via increased allopregnanolone, disrupting ovulation without altering cycle length or regularity. This points to a potential medication-specific impact on natural fertility.	SSRI, TCA	Fluoxetine, Sertraline, Citalopram, Paroxetine
Ekinci and Gunes 2019 [23]	Case report	This case highlights the first reported instance of sertraline-induced hyperprolactinemic amenorrhea in a 17-year-old girl, with symptoms resolving after discontinuation of Sertraline. This case analyzes how Sertraline and other SSRIs may elevate prolactin levels by stimulating serotonergic receptors or inhibiting dopaminergic pathways in the hypothalamus. While endocrine side effects are rare, this case underscores the importance of monitoring menstrual changes in adolescents on SSRIs.	SSRI	Sertraline
Gozdzik, et al. 2024 [24]	Experimental study (animal study)	This study demonstrated that Norfluoxetine (NFLU), a metabolite of Fluoxetine, acts as an endocrine-disrupting chemical in Mytilus trossulus (mussels) by altering gonadal serotonin concentrations and gene expression related to reproduction. NFLU accelerated oocyte maturation and gamete release in females while inhibiting spermatogenesis and delaying spawning in males, ultimately disrupting synchronized reproduction. These sex-specific effects occurred at environmentally relevant concentrations, suggesting that even low levels of SSRIs may impair reproductive success in non-target marine invertebrates.	SSRI	Norfluoxetine, Fluoxetine
Isik et al. 2022 [25]	Case report	Vortioxetine is a multimodal antidepressant generally considered safe, but this case highlights a potential adverse effect of amenorrhea in a 36-year-old woman with no prior menstrual irregularities. After three months of treatment and absent menses, her menstrual cycle resumed within two weeks of discontinuing the medication, suggesting a likely causal relationship.	Atypical	Vortioxetine
Junquiera et al. 2023 [26]	Literature review	This review found that drug-induced hyperprolactinemia commonly presents with menstrual disturbances, breast and lactation disorders, sexual dysfunction, and, less frequently, infertility and other reproductive symptoms. These effects were most often observed in women treated with antipsychotics, antidepressants, and other prolactin-elevating medications. The findings underscore the broad impact of elevated prolactin on reproductive and endocrine health.	TCA, SSRI	Unspecified antidepressants
Kumsar et al. 2014 [27]	Observational study (cohort study)	This study reviewed 52 premenopausal women (aged 25–46) diagnosed with major depression and found that premenopausal women with major depression had lower total and bioavailable testosterone levels compared to healthy controls, which normalized after six weeks of sertraline treatment - supporting a potential role for testosterone in mood regulation. However, despite improved depression scores and hormonal changes, sexual dysfunction persisted, likely due to SSRI-related serotonergic effects on dopaminergic pathways. These findings highlight the need to consider alternative or adjunctive treatments for SSRI-induced sexual side effects in women.	SSRI	Sertraline
Lee and Chung 2024 [28]	Case study	This case report describes a 25-year-old woman treated with high-dose vortioxetine for depression, developed abnormal bleeding, including tarry stools, ecchymosis, and massive uterine bleeding. The findings highlight a potential bleeding risk associated with vortioxetine, emphasizing the need for careful monitoring in menstruating women and thorough medication reviews in patients presenting with unexplained bleeding.	Atypical	Vortioxetine
Maden et al. 2019 [29]	Case report	This case describes a 43-year-old woman who developed amenorrhea after long-term treatment with escitalopram for anxiety, despite having no prior menstrual irregularities and normal prolactin levels. The case suggests escitalopram may contribute to menstrual disruption via increased ACTH rather than hyperprolactinemia. Clinicians should consider SSRI-related endocrine effects when evaluating unexplained amenorrhea.	SSRI	Escitalopram
Nguyen et al. 2017 [30]	Experimental study (animal study)	This study found that fluoxetine disrupts key signaling pathways in ovarian granulosa cells, including dose-dependent inhibition of cAMP accumulation, reduced ATP production, and elevated intracellular calcium levels. At higher concentrations, fluoxetine also decreased cell viability, suggesting potential cytotoxicity. These results indicate that fluoxetine may impair ovarian cell function and reproductive hormone signaling, even at clinically relevant doses.	SSRI	Fluoxetine
Romero-Reyes et al. 2016 [31]	Experimental study (animal study)	This study examined the effects of fluoxetine on reproductive development in prepubertal female rats and found that FLX elevated ovarian serotonin levels, altered ovarian structure, and disrupted follicular development. Specifically, it reduced ovulation while increasing the number of small and medium follicles. These findings suggest that fluoxetine may delay or disrupt puberty onset by modulating serotonergic activity along the hypothalamus-pituitary-ovarian axis.	SSRI	Fluoxetine
Schroeder et al. 2013 [32]	Case report	This case report details a 28-year-old woman who developed galactorrhea and hyperprolactinemia after four weeks of treatment with mirtazapine, an atypical antidepressant. Despite initial normal prolactin levels, elevation occurred after 12 days, and symptoms resolved upon switching to escitalopram. The findings suggest that mirtazapine may induce prolactin dysregulation via serotonergic mechanisms, challenging prior assumptions about its minimal impact on prolactin and aligning with growing evidence of antidepressant effects on reproductive hormones.	Atypical	Mirtazapine
Spadi et al. 2024 [33]	Observational study	This study examined 27 healthy controls, matched by age and contraceptive use, alongside premenopausal female patients aged 18 years and older with mood disorders receiving psychotropic treatment. Exclusions included pregnancy, serious acute somatic illness, or a comorbid mental disorder not considered primary. Significant menstrual cycle–related variations in antidepressant pharmacokinetics were observed, with serum concentrations of Bupropion’s active metabolite lowest during menstruation and highest in the late luteal phase, and similar fluctuations noted for venlafaxine. These results highlight the influence of hormonal shifts on drug metabolism and support the need for personalized, cycle-aware treatment strategies in women.	Atypical, SSRI, SNRI	Bupropion, venlafaxine, sertraline
Sun et al. 2024 [34]	Narrative review	This review examines the central role of estrogen and its receptors in the neuroendocrine, neuroplastic, and neuroinflammatory pathways that shape depression across the female reproductive cycle. Evidence suggests that estrogen can modulate antidepressant response, and that fluoxetine itself interacts with estrogen signaling in complex, dose-dependent ways that may influence both mood regulation and reproductive function. These findings underscore the need for hormone-informed, personalized approaches to depression treatment in women.	SSRI	Fluoxetine
Yakimova et al. 2020 [35]	Observation study	This study analyzed women aged 18-50 years, with regular menstrual cycles (21-35 days), with major depressive disorder. SSRI therapy markedly lessened premenstrual syndrome symptoms, with the bulk of improvement, especially in mood swings, anxiety, and irritability, occurring within the first three months. Somatic complaints such as breast tenderness, bloating, and headaches subsided more slowly and often persisted, underscoring that antidepressants differentially affect psychological and physical facets of the reproductive cycle. These findings offer direct evidence that SSRIs modulate cycle-related symptomatology, but their impact on somatic manifestations may be incomplete.	SSRI	Citalopram, fFuoxetine, Paroxetine, Escitalopram

Characteristics of the Reviewed Studies

The distribution of studies targeting each class of antidepressants is outlined in Table 2 and underscores the uneven distribution of studies across these four drug classes. The most frequently studied SSRI was fluoxetine (n=6) [22, 24, 30, 31, 34, 35], followed by sertraline (n=4) [22, 23, 27, 33], escitalopram (n=2) [29, 35], citalopram (n=2) [22, 35], and paroxetine (n=2) [22, 35] (Table 2). Among atypical antidepressants, bupropion (n=2) [21, 33], mirtazapine (n=1) [32], and vortioxetine (n=2) [25, 28] were represented. One study examined venlafaxine, an SNRI (n=1) [33], and two studies included a TCA, although the specific agent was not identified in either study (n=2) [22, 26]. One study did not specify the antidepressants used by participants in the analysis [20]. Several studies evaluated multiple classes, most commonly SSRIs with TCAs [22, 26] and SSRIs with SNRIs [33]. Experimental studies primarily used animal models to examine the effects of antidepressants on reproductive physiology, focusing on mechanisms such as serotonergic signaling, hormonal regulation, and ovarian structure [24, 30, 31]. Across observational studies, menstrual patterns, ovulation, and hormone levels were the most reported outcomes.

**Table 2:** Matrix of studies categorized by antidepressant class The table shows the uneven distribution of studies targeting these four classes of drugs. SSRIs accounted for the majority of investigations, with fewer studies examining atypicals, TCAs, and SNRIs. Several studies assessed more than one class, most commonly SSRIs with TCAs. SSRIs - selective serotonin reuptake inhibitors; TCAs - tricyclic antidepressants; SNRIs - selective norepinephrine reuptake inhibitors

Articles	Atypical	SNRI	SSRI	TCA	Unspecified
Bleil et al. 2013 [20]					X
Çam and Bilgin 2015 [21]	X				
Casilla-Lennon et al. 2016 [22]			X	X	
Ekinci and Gunes 2019 [23]			X		
Gozdzik, et al. 2024 [24]			X		
Isik et al. 2022 [25]	X				
Junquiera et al. 2023 [26]			X	X	
Kumsar et al. 2014 [27]			X		
Lee and Chung 2024 [28]	X				
Maden et al. 2019 [29]			X		
Nguyen et al. 2017 [30]			X		
Romero-Reyes et al. 2016 [31]			X		
Schroeder et al. 2013 [32]	X				
Spadi et al. 2024 [33]	X	X	X		
Sun et al. 2024 [34]			X		
Yakimova et al. 2020 [35]			X		

Hormonal Regulation and HPO Axis Modulation

Five studies identified associations between serotonergic antidepressants and alterations in endocrine signaling, including testosterone regulation, HPO axis activity, and gonadotropin responsiveness [27, 29-31, 34]. Clinical data indicated normalization of testosterone levels following SSRI treatment in premenopausal women with major depressive disorder, despite persistence of sexual dysfunction [27]. Additional reports documented menstrual irregularities with endocrine changes, such as elevated adrenocorticotropic hormone (ACTH) and resolution of amenorrhea after SSRI discontinuation [29]. Experimental studies demonstrated fluoxetine-induced suppression of FSH-mediated signaling in granulosa cells and increased serotonergic activity within ovarian and pituitary tissues [30, 31]. Estrogen-serotonin interactions were also shown to modulate hypothalamic gonadotropin-releasing hormone (GnRH) expression and pituitary hormone release under SSRI exposure [34].

Menstrual Irregularities and Amenorrhea

Clinical evidence linked serotonergic antidepressants to menstrual disturbances. Case reports described SSRI-induced amenorrhea with restoration of menses after drug discontinuation or substitution [23, 29]. In one case, prolonged escitalopram use caused amenorrhea with elevated ACTH and normal prolactin; menses resumed after switching therapy [29]. Another report documented sertraline-induced amenorrhea in an adolescent without hyperprolactinemia resolving after discontinuation [23]. In a prospective cohort of women with premenstrual syndrome and depression, somatic symptoms such as bloating and mastalgia persisted during SSRI treatment despite mood improvement [35]. Pharmacokinetic analysis revealed antidepressant plasma concentrations of bupropion and venlafaxine fluctuated across the menstrual cycle, which was attributed to hormone-mediated changes in metabolism and drug distribution [33]. Plasma concentrations of these drugs were lowest during menstruation and highest during the late luteal phase [33]. Notably, venlafaxine levels showed a secondary peak during menses and were positively correlated with 17-α-hydroxyprogesterone concentrations at ovulation [33].

Preclinical findings supported these observations. Fluoxetine-treated rats exhibited serotonin accumulation in ovarian and pituitary tissues, increased small and atretic follicles, and reduced ovulatory output despite unchanged LH and FSH concentrations [31]. In vitro, fluoxetine exposure suppressed FSH-induced cyclic adenosine monophosphate (cAMP) signaling in granulosa cells and disrupted intracellular calcium and adenosine triphosphate (ATP) homeostasis, indicating impaired gonadotropin responsiveness [30].

Reproductive and Fertility Outcomes

Seven studies examined fertility-related outcomes, reporting alterations in ovulation, folliculogenesis, and conception probability [22, 27, 29-31, 34, 35]. Two studies associated antidepressant exposure with reduced probability of conception per cycle, with one study specifically linking SSRI use to impaired fertility [22, 30]. Experimental work demonstrated fluoxetine-related disruptions in ovarian development and timing of ovulation in prepubertal rats [31] as well as impaired granulosa cell function in vitro [30]. SSRI-induced sexual dysfunction persisted despite normalization of testosterone levels following antidepressant therapy, with parameters including libido, arousal, and orgasm remaining impaired [27].

Hormonal Dysregulation

Endocrine alterations were described in seven studies [21, 27, 29, 31, 32, 34, 35]. Clinical cases included escitalopram-associated amenorrhea with ACTH elevation [29], with experimental findings showing that estrogen depletion combined with corticosterone exposure disrupted GnRH signaling and estrous cyclicity, which was reversed with estrogen replacement [34]. Two case reports documented hyperprolactinemia: one associated with mirtazapine, accompanied by galactorrhea, mastodynia, and edema that resolved after drug discontinuation [32], with another involving bupropion that was attributed to disruption of dopamine-mediated prolactin regulation [21]. In a prospective study, somatic premenstrual symptoms such as breast tenderness and bloating persisted despite improved mood over six months of SSRI therapy [35].

Discussion

Findings from this scoping review illustrate the multifaceted, sex-specific interactions between antidepressant pharmacology and reproductive endocrinology. Evidence from preclinical, clinical, and pharmacokinetic studies supports two complementary biological pathways: central hypothalamic pituitary modulation [21, 22, 24, 29-33] and peripheral ovarian disruption [29]. Through these mechanisms, antidepressants, particularly serotonergic agents, may influence menstrual regularity, ovulation, steroid hormone production, and overall fertility potential. Consistent signals across diverse study designs reinforce the biological plausibility of menstrual disturbances, hormonal alterations, and ovulatory dysfunction, underscoring the importance of reproductive health considerations when prescribing antidepressants to individuals of reproductive age.

Preclinical work with fluoxetine provides the clearest example of a peripheral mechanism. In prepubertal rats, fluoxetine exposure produced elevated intra‑ovarian serotonin concentrations, increased counts of small and atretic follicles, oocyte fragmentation, and a reduction in total ova shed despite unchanged gonadotropin levels [31]. Parallel in vitro experiments showed that fluoxetine impaired granulosa‑cell responsiveness to FSH by lowering intracellular cAMP and adenosine triphosphate (ATP) and inducing a sustained Ca²⁺ influx consistent with diminished steroidogenesis and follicular maturation [30].

Central neuroendocrine effects appear equally relevant. Clinically, escitalopram-induced amenorrhea with ACTH elevation resolved after discontinuation and switching to trazodone, without hyperprolactinemia or gonadotropin changes [29]. Similarly, sertraline initiation was temporally linked to menstrual suppression in an adolescent [23]. These findings implicate serotonergic activation of the HPA axis, rather than dopaminergic or prolactin pathways, as a potential driver of SSRI-related menstrual disturbances.

Antidepressant treatment can normalize pre‑existing hormonal deficits yet leave functional complaints unresolved. Depressed women displayed significantly lower total and bioavailable testosterone than controls; sertraline restored androgen levels after six weeks, but sexual dysfunction persisted [27]. Likewise, in women with premenstrual syndrome (PMS) comorbid with major depressive disorder, SSRI therapy reduced psychological symptom burden, yet somatic symptoms such as breast tenderness and bloating remained common at six months [35]. Case reports of multimodal vortioxetine therapy have linked the drug to amenorrhea and abnormal uterine bleeding, suggesting that even agents with mixed pharmacology warrant reproductive monitoring [28].

Drug-specific trends were notable: fluoxetine demonstrated the strongest evidence for direct ovarian effects; escitalopram was linked to HPA-mediated amenorrhea; sertraline appeared to produce milder endocrine changes; and venlafaxine and bupropion showed menstrual phase-dependent pharmacokinetics. These observations underscore heterogeneity in reproductive risk across antidepressant classes and highlight the need for individualized monitoring and therapy in reproductive-age patients. As summarized in Table 3, antidepressants demonstrate a range of effects on reproductive hormones and axis regulation, with mechanisms spanning cellular, endocrine, and systemic levels. 

**Table 3:** Reproductive effects of antidepressants - summary of included studies SSRI - selective serotonin reuptake inhibitors; GnRH - gonadotropin-releasing hormone; LH - luteinizing hormone; FSH - follicle-stimulating hormone; HPO - hypothalamic-pituitary-ovarian; HPA - hypothalamic-pituitary-adrenal; cAMP - cyclic adenosine monophosphate; 5-HTR-1 - 5-hydroxytryptamine receptor; ACTH - adrenocorticotropic hormone; 5-HT - 5-hydroxytryptamine; HPG - hypothalamic-pituitary-gonadal

Antidepressant	Reproductive and/or Hormonal effect	HPO vs. HPA
General overview SSRI	↑ Allopregnanolone → suppresses GnRH release → ↓ LH/FSH → impaired ovulation [22]. Findings suggest class-wide SSRI effect on the HPO axis, mediated through allopregnanolone-induced suppression of GnRH and gonadotropin release [22].	HPO
Fluoxetine (SSRI)	Cellular level: Inhibits cAMP accumulation in granulosa cells at low concentrations, suggesting impaired granulosa cell function and follicular development [30]. Endocrine level: ↑ ovarian serotonin and atretic follicles, leading to ↓ estradiol and ↑ prolactin [31]. These findings suggest fluoxetine disrupts the HPO axis both locally, through impaired granulosa cell signaling, and systemically, via serotonin-mediated suppression of estradiol and prolactin elevation [30-31].	HPO
Sertraline (SSRI)	Associated with ↑ FSH and ↑ premenstrual estradiol indicating alterations in gonadotropin output and ovarian hormone patterns [33]. These findings suggest that sertraline may modulate ovarian steroidogenesis through HPO-axis dysregulation, with enhanced gonadotropin stimulation contributing to changes in estradiol levels during the luteal phase [33].	HPO
Norfluoxetine (SSRI) - animal model	Stimulates gonadal serotonin → induces oocyte maturation and gamete release; alters 5-HTR1 gene expression (↑ females and ↓ males) [24]. These findings suggest that norfluoxetine may directly modulate gonadal serotonergic signaling within the HPO axis, influencing oocyte development and gamete release [24].	HPO
Escitalopram (SSRI)	Linked to amenorrhea accompanied by elevated ACTH levels, indicating a potential association between antidepressant exposure and menstrual dysfunction [29]. These findings suggest that escitalopram may exert reproductive effects through HPA-axis activation, with secondary suppression of the HPO axis via stress-related hormonal pathways. This highlights the interplay between stress regulation and reproductive function in antidepressant-associated menstrual changes [29].	HPA (secondary HPO)
Bupropion (atypical)	Expected ↓ in prolactin via dopamine-mediated inhibition of pituitary prolactin release (HPO axis) [33]. Paradoxical cases of hyperprolactinemia have been reported, potentially due to dopamine receptor downregulation with chronic exposure [21].	HPO
Mirtazapine (atypical)	Mirtazapine-induced galactorrhea with elevated thought to be mediated via serotonin-driven prolactin release through hypothalamic 5-HT receptors [32]. Although earlier reports suggested mirtazapine does not cause hyperprolactinemia, this case highlights a possible paradox, indicating that serotonin-mediated mechanisms may still be relevant in select individuals [32].	HPO
Venlafaxine (SNRI)	Associated with ↑ 17 a-hydroxyprogesterone levels, with a positive correlation observed during ovulation [33]. This correlation suggests possible modulation of ovarian steroidogenesis/HPG axis potentially altering ovulatory dynamics [33].	HPO

At the class level, SSRIs are associated with suppression of GnRH release through allopregnanolone, resulting in reduced LH/FSH output and impaired ovulation [22]. Fluoxetine further illustrates this disruption, with granulosa cell inhibition of cAMP accumulation at low concentrations [30], increased ovarian serotonin and atretic follicles, decreased estradiol, and elevated prolactin [31]. These findings highlight both local and systemic effects on the HPO axis. In contrast, sertraline has been linked to increased FSH and premenstrual estradiol, suggesting altered gonadotropin stimulation and luteal phase estradiol dynamics [33]. Evidence also suggests direct serotonergic effects at the gonadal level, as shown by norfluoxetine in animal models, which stimulated oocyte maturation, gamete release, and sex specific alternation in 5-hydroxytryptamine receptor 1 (5-HTR1) gene expression [24].

Beyond the HPO axis, certain antidepressants appear to involve HPA activation with secondary reproductive consequences. Escitalopram has been linked to amenorrhea with elevated ACTH, indicating menstrual dysfunction through stress-related hormonal pathways [29]. Similarly, atypical antidepressants such as bupropion and mirtazapine demonstrate variable effects on prolactin: bupropion is generally associated with decreased prolactin through dopaminergic mechanisms, though paradoxical hyperprolactinemia has been reported [21,33], while mirtazapine has been implicated in galactorrhea with elevated prolactin, likely via serotonergic pathways [32]. Finally, SNRIs such as venlafaxine have been associated with increased 17-a-hydroxyprogesterone, correlating with ovulation and suggesting possible modulation of ovarian steroidogenesis [33].

**Figure 2:**
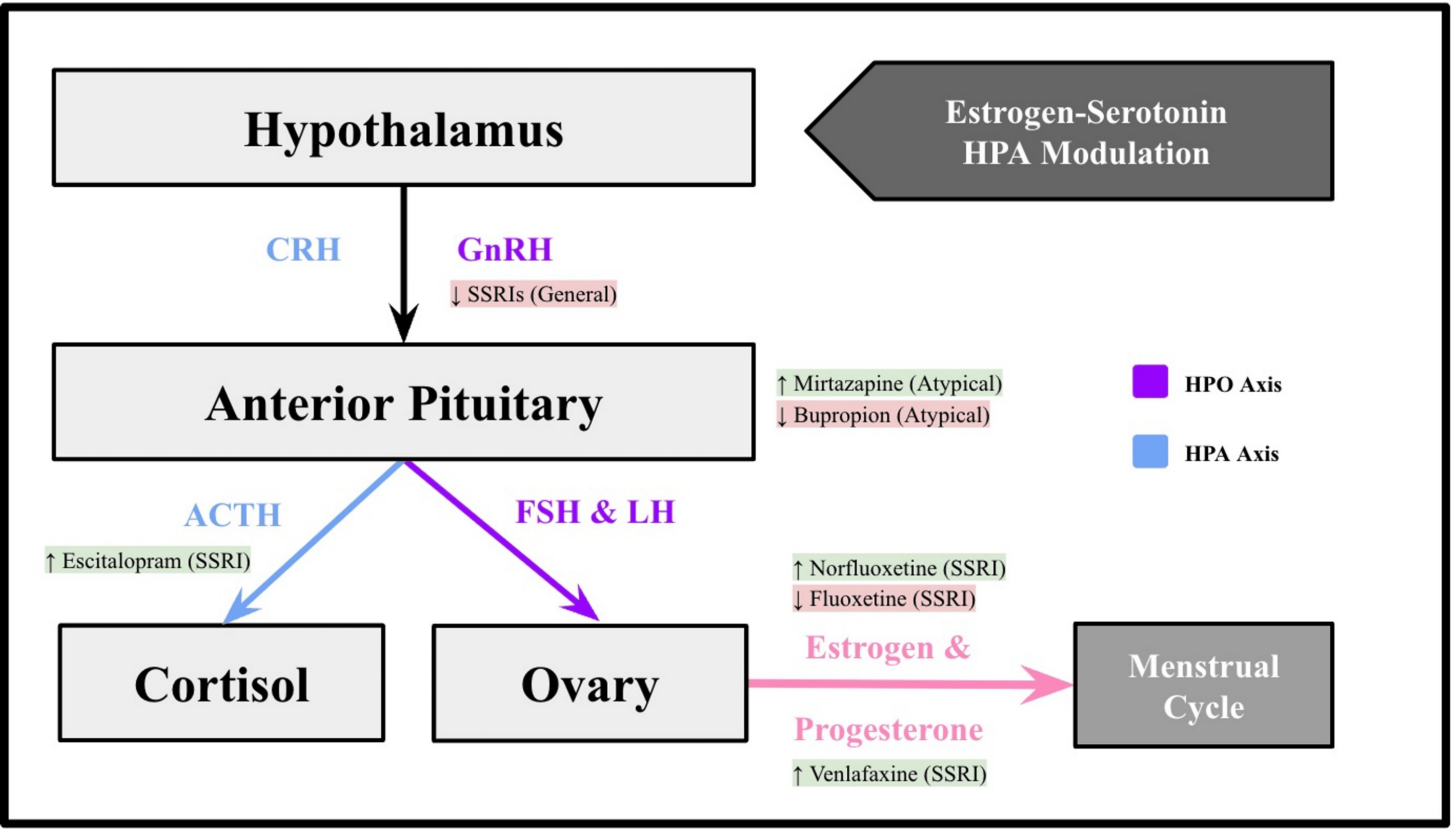
Hypothalamic-pituitary-ovarian (HPO) and hypothalamic-pituitary-adrenal (HPA) axis crosstalk CRH - corticotropin-releasing hormone; GnRH - gonadotropin-releasing hormone; SSRI  - selective serotonin reuptake inhibitors; ACTH - adrenocorticotropic hormone; LH - luteinizing hormone; FSH - follicle-stimulating hormone; HPO - hypothalamic-pituitary-ovarian; HPA - hypothalamic-pituitary-adrenal

Taken together, these mechanisms indicate that antidepressant effects on reproductive health extend beyond mood regulation and involve multi-level disruption of endocrine signaling. By acting through the HPO and HPA axes, as well as directly on ovarian and pituitary pathways, these agents may alter ovulation, gonadotropin release, and prolactin regulation in ways that are clinically significant (Figure 2).

These findings reinforce the importance of individualized hormonal monitoring in reproductive-age women undergoing SSRI treatment, especially in the presence of mood instability, menstrual changes, or persistent sexual dysfunction. Given the widespread use of these medications among women of childbearing age, clinicians should obtain baseline and follow‑up menstrual histories, consider drug substitution when amenorrhea or heavy bleeding arises, and counsel patients on potentially reversible fertility effects. Future research should prioritize longitudinal, hormone‑monitored cohorts, pharmacokinetic modelling across the menstrual cycle, and systematic inclusion of reproductive endpoints in psychopharmacology trials.

Limitations

Several limitations should be considered when interpreting the findings of this review. Much of the mechanistic evidence derives from animal models and in vitro studies, which, while valuable for elucidating cellular and neuroendocrine pathways, may not fully capture the complexity of human reproductive physiology. Many clinical investigations were limited to case reports or small observational cohorts, some containing a single subject, often with brief follow-up periods that restricted generalizability. In particular, several case reports described only a single patient, which further constrains the ability to draw broader conclusions or identify consistent patterns. Hormonal monitoring was inconsistent, with numerous studies relying on self-reported menstrual changes rather than objective endocrine assays. Use of oral contraceptives was rarely reported or controlled for, despite their potential to mask or confound antidepressant-related menstrual effects, particularly in individuals with underlying or subclinical cycle abnormalities. Pregnancy history was not included in these studies, which restricted the ability to observe any effect of prior pregnancies on antidepressant use. Finally, few studies accounted for the menstrual phase in data collection or analysis, despite well-established cyclical variations in hormone levels and drug metabolism.

Methodological limitations of the review itself must also be acknowledged. The search was restricted to a limited number of databases, which may have led to the omission of relevant studies indexed elsewhere. Although search terms were designed to capture a broad range of reproductive and hormonal outcomes, they may not have encompassed all terminology used in the literature. Restricting inclusion to peer-reviewed studies published in English between 2010 and 2025 introduces the potential for language bias and for publication bias, as unpublished or non-English studies with null findings may have been excluded. Finally, the inclusion of heterogeneous study designs and outcome measures, while appropriate for a scoping review, limits the ability to draw definitive conclusions regarding causality or comparative effectiveness.

Together, these limitations underscore the need for rigorously designed prospective studies that incorporate standardized hormone assays, phase-specific sampling, and stratification by contraceptive use, along with systematic reviews that include broader database coverage and multilingual sources. At the same time, individual case reports, despite their inherent constraints, provide exploratory insights that can generate hypotheses and highlight clinically relevant signals that warrant further investigation.

## Conclusions

This scoping review highlights the potential for antidepressants to impact female reproductive physiology through both central neuroendocrine disruption as well as direct ovarian effects. Evidence from animal models, in vitro experiments, clinical case reports, and cohort studies demonstrates associations between serotonergic antidepressant use and menstrual irregularities, amenorrhea, altered ovulation, and hormonal fluctuations. While antidepressants remain essential in the management of mood disorders, these findings highlight the importance of monitoring reproductive health in women of reproductive age. Importantly, resolution of mood symptoms does not always correspond with restoration of menstrual or hormonal function, suggesting the need for more nuanced patient follow-up. Future research should prioritize prospective studies with standardized tracking of hormonal parameters, menstrual patterns, and contraceptive use to clarify the frequency, mechanisms, and clinical relevance of these effects. Clinicians should maintain a high index of suspicion for reproductive side effects, counsel patients appropriately, and consider individualized approaches that integrate both mental and reproductive health goals.
